# Overexpression of DWF1 Enhances Low-Nitrogen Stress Tolerance in Potato Plants

**DOI:** 10.3390/ijms26094374

**Published:** 2025-05-04

**Authors:** Zi Li, Zhuangyan Li, Yifei Lu, Bi Ren, Fuchun Zeng, Shimin Yang, Liming Lu, Liqin Li

**Affiliations:** College of Agronomy, Sichuan Agricultural University, Chengdu 611130, China; lizi824842227@163.com (Z.L.); lizhuangyan1014@163.com (Z.L.); sarklu@126.com (Y.L.); renbi1232021@163.com (B.R.); zengfuchun78@163.com (F.Z.); yangshimin1@163.com (S.Y.); luliming@sicau.edu.cn (L.L.)

**Keywords:** transgenic potato, *StDWF1*, nitrogen metabolism, stress tolerance, hormonal regulation, gene expression

## Abstract

Nitrogen is an important nutrient required for plant growth and development, but most of the time plants face nitrogen deficiency, all it is important to study the mechanism of low nitrogen tolerance in plants. This study addresses this gap by investigating the role of the *StDWF1* gene through the generation and analysis of transgenic potato lines overexpressing *StDWF1* (OE1, OE2, OE3). Exogenous BL treatment showed that the *StDWF1* gene responded to oleuropein lactone. Phenotypic assessments under normal nitrogen (NN) and low nitrogen (LN) conditions demonstrated that OE2 consistently outperformed WT, showing a 43% increase in root vitality and a 23% retention of chlorophyll under LN. Additionally, OE2 transgenics accumulated significantly higher levels of nitrate nitrogen (64.1% increase) and ammonium nitrogen (53% increase) compared to WT. Enzymatic assays further confirmed elevated activities of glutamine synthetase and nitrate reductase in both OE1 and OE2 lines. Hormone analyses showed that BL content of *StDWF1* overexpression lines was significantly increased under LN conditions, higher Oleandrin lactone (BL) content of OE2 improved plant stress tolerance, and WT was more affected by low nitrogen stress than OE2, resulting in higher levels of stress hormones than OE2. Temporal gene expression analysis showed significant upregulation of key nitrogen metabolism-related genes (*NR*, *NiR*, *AT*, *NRT2.1*) in OE2, with *StDWF1* expression reaching 79% higher than WT at 3 h. Protein–protein interaction assays, including yeast two-hybrid and BiLC assays, verified the interaction between *StDWF1* and *StGRP1*, suggesting the existence of a functional network to enhance low-nitrogen tolerance in potato plants. In conclusion, these findings suggest that overexpression of *StDWF1* significantly enhances low-nitrogen tolerance in transgenic potato lines, providing a promising strategy for improving crop performance under nitrogen-limited conditions.

## 1. Introduction

Nitrogen (N) is an essential macronutrient for plant growth and development, playing a key role in synthesizing fundamental biomolecules such as proteins, chlorophyll, nucleic acids, and enzymes [1,2,3]. These molecules are indispensable for vital physiological processes, including photosynthesis, cellular metabolism, and stress responses [4]. Despite the abundance of nitrogen in the atmosphere, most of it exists in an inert form (N_2_) that plants cannot directly utilize [5]. Consequently, plants rely on nitrogen in more bioavailable forms, such as nitrate (NO_3_⁻) and ammonium (NH_4_⁺), acquired from the soil through root absorption [6]. In agricultural systems, nitrogen availability is often a limiting factor for crop productivity. Plants cultivated in nitrogen-deficient soils exhibit reduced growth rates, decreased chlorophyll content, and lower yields [7,8]. The conventional remedy for nitrogen deficiency is the application of synthetic nitrogen fertilizers. While effective in increasing crop yields, excessive dependence on nitrogen fertilizers has led to serious environmental issues, including soil degradation, eutrophication of water bodies, and increased greenhouse gas emissions during fertilizer production and use [9,10]. Moreover, the economic burden of rising fertilizer costs is a source of concern for farmers worldwide [11]. Therefore, exploring the physiological response and adaptive mechanism of potato under low nitrogen stress, developing the genetic potential for efficient nitrogen utilization, and breeding potato varieties that are more tolerant to low nitrogen stress will help to improve the yield and quality of potato in nitrogen-deficient soils, reduce the loss of nitrogen fertilizers and environmental pollution, and promote the sustainable development of agriculture.

Plant hormones exhibit a central role in regulating nitrogen uptake, transport, and assimilation [12]. Various hormones including auxins, cytokinins, abscisic acid (ABA), ethylene, gibberellins (GA), jasmonic acid (JA), and brassinosteroids (BR) influence nitrogen metabolism at different levels [13]. For example, auxins shape root architecture to enhance nitrogen acquisition, while cytokinins help balance nitrogen distribution between roots and shoots [14]. Under nitrogen deficiency, ABA and JA contribute to stress responses by stimulating root growth and improving nitrogen uptake [15]. Recent studies indicate that BRs play a key role in regulating root elongation and nitrate transporter expression in response to nitrogen availability [16]. This complex interaction between nitrogen and hormone signaling provides promising avenues for improving low-nitrogen tolerance in plants. BRs, a group of plant-specific steroid hormones, are essential for growth, development, and stress adaptation [16]. They significantly impact root architecture, a crucial factor in water and nutrient uptake [13]. Research on *Arabidopsis thaliana* and rice has shown that BR signaling influences root elongation and branching, particularly under nitrogen-deficient conditions [17,18]. For instance, the BRASSINOSTEROID SIGNALING KINASE3 (*BSK3*) gene is involved in primary root elongation under mild nitrogen deficiency [19]. BRs also regulate nitrogen transporters and enzymes involved in nitrogen metabolism [20,21]. Specifically, BR signaling modulates NRTs such as *AtNPF6.3*, facilitating nitrate uptake and movement within the plant [22]. In rice, BR signaling is linked to spikelet formation and grain yield in response to nitrogen fertilization, emphasizing its role in balancing nitrogen acquisition with plant growth and productivity [23].

In recent years, the interaction of oleaginous sterols (BR) and their signaling pathways with the response to low nitrogen stress has attracted much attention [24]. The group identified the key gene DWF1 for potato BR biosynthesis for the first time, and found that this gene catalyzes the synthesis of BR and other related substances affecting the growth and development of potato [25]. DWF1 is a class of FAD-dependent oxidoreductases with highly conserved functions. In Arabidopsis, DWF1/DIM mutations block BR synthesis, leading to plant dwarfism [26]; maize DWF1 RNAi lines show growth inhibition due to BR deficiency [27]. Collectively, these observations indicate that DWF1, a pivotal gene in BR biosynthesis, may play a role in plant morphogenesis and stress adaptation. This study focused on the StDWF1 gene and its role in improving potato (Solanum tuberosum) tolerance to low nitrogen stress. The aim of this study was to investigate phenotypic and physiological changes in plants of WT and OE lines grown under LN and NN conditions, as well as the expression of genes related to GS and NR enzyme activities and nitrogen metabolism, by constructing transgenic potato lines overexpressing *StDWF1*, and to investigate *StDWF1* protein interactions to understand their role in nitrogen signaling pathways. This research provides an important insight into the molecular mechanisms behind low-nitrogen tolerance in potato plants and lays the foundation for the development of crop varieties that can thrive in nitrogen-limited environments.

## 2. Results

### 2.1. StDWF1 Expression and Phenotypic Characterization of Transgenic Lines

**Figure 1:**
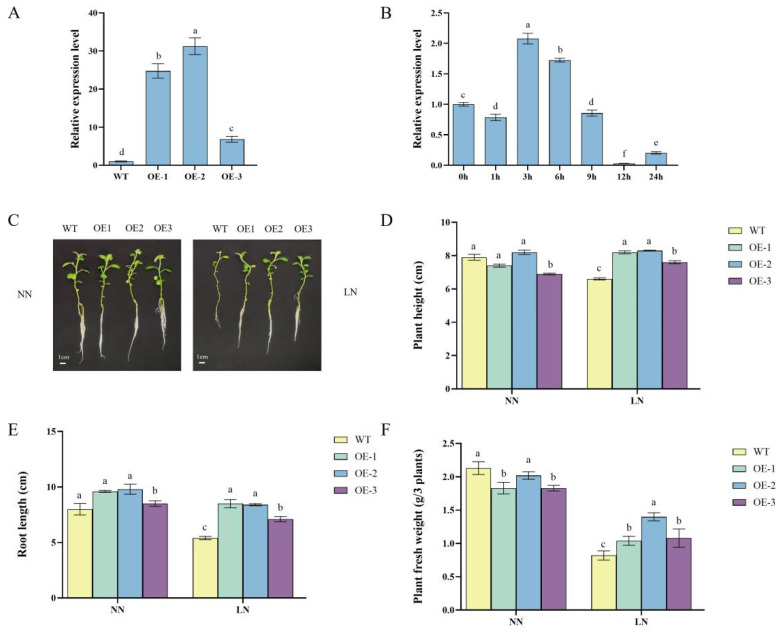
Overexpression of *StDWF1* enhances low nitrogen tolerance in plants. (**A**) Quantitative RT-PCR analysis of *StDWF1* expression in wild type (WT) and *StDWF1* overexpression lines (OE1, OE2, OE3). (**B**) Time-course analysis of *StDWF1* expression in WT plants under exogenous BR treatment. (**C**) Phenotypic analysis. (**D**) Plant height comparison under normal nitrogen (NN) and low nitrogen (LN) conditions for WT and overexpression lines. (**E**) Root length comparison under NN and LN conditions. (**F**) Fresh weight of plants under NN and LN conditions. Data represent the mean ± SE for WT and overexpression lines (*n* = 3). Different letters indicate statistically significant differences according to Student’s *t* test (*p* < 0.05).

The expression analysis of *StDWF1* in transgenic lines demonstrated significant differences. The OE2 line showed the highest expression level, which was approximately 30-fold higher than WT, followed by OE1 with a 23-fold increase and OE3 with a modest 5.8-fold increase compared to WT (Figure 1A). The temporal expression pattern of *StDWF1* was examined in WT plants following treatment with 500 nM BL, with relative gene expression monitored over time. The results revealed a dynamic regulatory response, with expression significantly increasing (*p* < 0.05) within the first hour of treatment. Expression peaked at 3 h, reaching 10% higher than the baseline at 0 h. Following this peak, expression progressively declined, showing a 24% reduction at 6 h relative to the 3 h peak. The decline continued at 9 h and beyond, reaching its lowest level at 12 h, where expression was 69-fold lower than at its peak (Figure 1B).

The phenotypic analysis of plants revealed significant differences between the WT and transgenic lines. Under NN conditions, the transgenic lines, particularly OE2 and OE1, displayed visibly enhanced shoot and root growth compared to WT (Figure 1C). OE2 exhibited the most robust phenotype, with longer roots and taller shoots compared to both WT and other transgenic lines. OE3 showed slightly less improvement compared to OE1 and OE2 but still outperformed WT. Under LN conditions, the differences between WT and transgenic lines became more pronounced. WT plants exhibited stunted growth with visibly shorter roots and shoots. In contrast, transgenic lines maintained relatively better growth, with OE2 once again demonstrating the most tolerance to nitrogen deficiency by sustaining greater shoot height and root length compared to WT. OE1 also showed improved growth under LN conditions but to a slightly lesser extent than OE2. OE3, while still better than WT, showed weaker performance compared to OE1 and OE2 under LN conditions.

Under LN conditions, the overexpression of *StDWF1* in transgenic lines significantly (*p* < 0.05) improved growth performance compared to the WT. Under LN, plant height in the OE2 line was significantly (*p* < 0.05) increased by 25.8% compared to WT, while OE1 also showed a notable improvement of 24.2% (Figure 1D). Root length was significantly (*p* < 0.05) enhanced in OE2 and OE1, with increases of 55.6% and 56%, respectively, compared to WT under LN (Figure 1E). Additionally, the fresh weight of plants under LN was markedly higher in OE2 and OE1, with OE2 showing an improvement of 70.7% and OE1 an increase of 26.8% relative to WT (Figure 1F).

### 2.2. Enhanced Nitrogen Accumulation and Metabolic Efficiency in stDWF1-Overexpressing Lines

**Figure 2:**
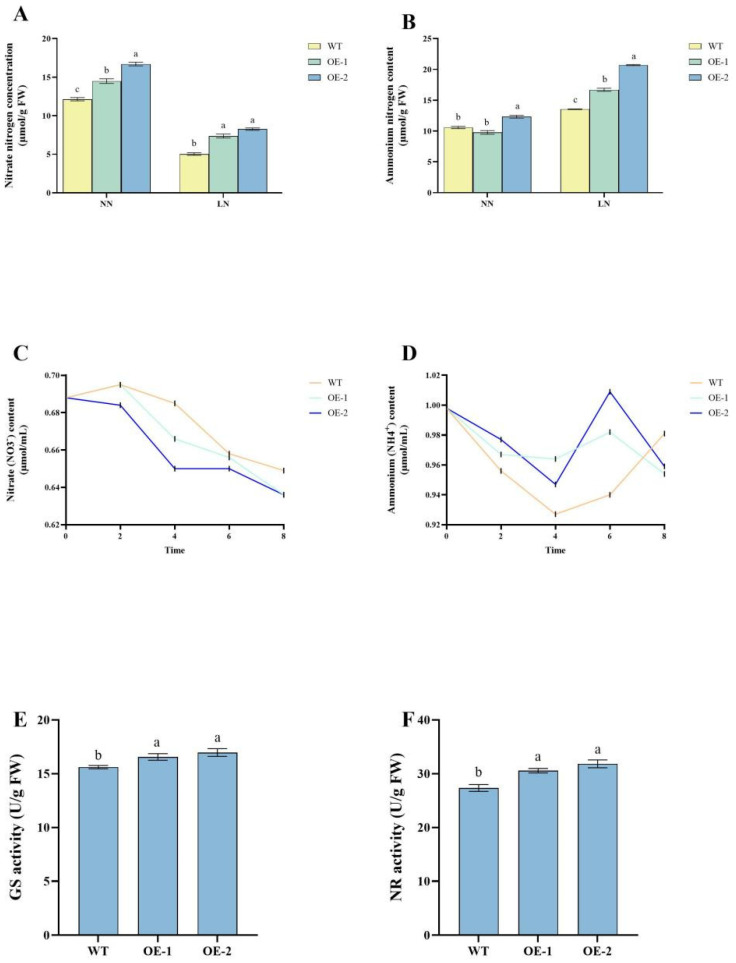
Impact of *StDWF1* overexpression on nitrogen metabolism in plants. (**A**) Nitrate content (NO_3_⁻) in WT and *StDWF1*-overexpressing plants (OE1, OE2). (**B**) Ammonium content (NH_4_⁺) in WT and *StDWF1*-overexpressing plants. (**C**) Time-course analysis of NO_3_⁻ content. (**D**) Time-course analysis of NH_4_⁺ content. (**E**) Glutamine synthetase (GS) activity. (**F**) Nitrate reductase (NR) activity. Bars represent means ± SE (*n* = 3). Different letters indicate statistically significant differences according to Student’s *t* test (*p* < 0.05).

The analysis of nitrate nitrogen content demonstrated that all transgenic lines accumulated significantly higher levels of nitrate nitrogen compared to the WT (*p* < 0.05). Under NN treatment, OE2 exhibited the highest nitrate nitrogen content, showing an approximate 40.1% increase over WT, followed by OE1, which displayed a 19.4% increase. In contrast, under LN conditions, OE2 demonstrated an even greater accumulation, with nitrate nitrogen levels increasing by 64.3% relative to WT, while OE1 showed a 46.3% increase (Figure 2A). Under NN treatment, the ammonium nitrogen content in OE2 was higher than that of WT, whereas other transgenic lines did not show significant differences compared to WT. However, under LN conditions, all overexpression lines exhibited significantly elevated ammonium nitrogen levels relative to WT. Among them, OE2 showed the highest accumulation, reaching 153% that of WT. Additionally, under LN conditions, OE2 accumulated approximately 109% more ammonium nitrogen than under NN treatment, whereas the ammonium nitrogen content in WT increased by only 28%. Notably, OE1 also exhibited significant ammonium nitrogen accumulation, comparable to OE2 (Figure 2B).

The nitrogen uptake rate was evaluated through a nitrogen depletion experiment. Nitrate concentration in the solution initially increased between 0 and 2 h before declining between 2 and 8 h. At 8 h, nitrate concentrations in OE1 and OE2 were lower than in WT, suggesting that the transgenic lines had a higher nitrate uptake capacity than WT (Figure 2C). Similarly, ammonium concentration in the solution declined from 0 to 4 h, then increased until 6 h, reaching its peak before dropping sharply between 6 and 8 h (Figure 2D). The ammonium concentration in the overexpression lines at 8 h was lower than that of WT, further indicating an enhanced nitrogen uptake capacity in transgenic plants compared to WT.

Plants subjected to nitrogen depletion conditions were further treated with depletion solution for 20 days. Under LN conditions, GS activity was significantly higher (*p* < 0.05) in OE1 and OE2 than in WT, with an 8.6% increase. However, there was no significant difference in GS activity between the two transgenic lines (Figure 2E). Similarly, NR activity was also significantly elevated (*p* < 0.05) in OE1 and OE2, with both lines showing an 11.6% increase compared to WT, indicating improved nitrate reduction efficiency (Figure 2F). The results of the study showed that the nitrogen assimilation capacity of the OE strain was higher than that of the WT strain.

### 2.3. Upregulation of Nitrogen Metabolism-Related Genes in Transgenic Lines

**Figure 3:**
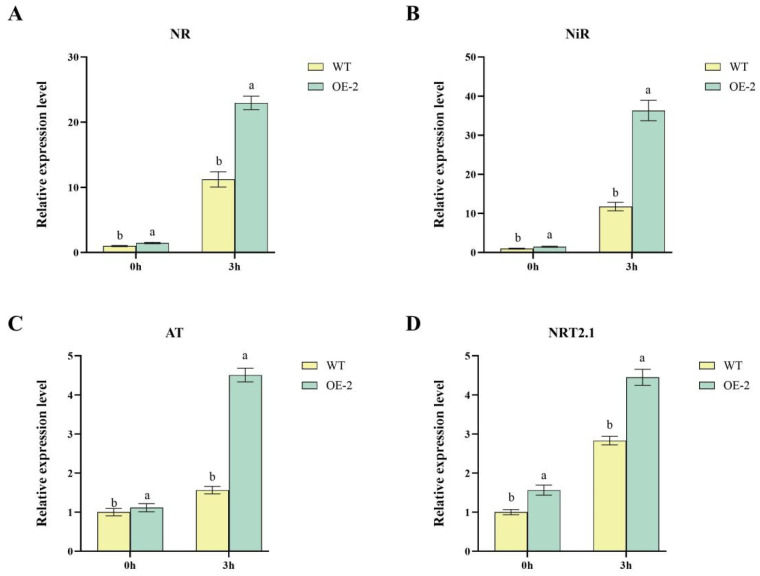
Expression analysis of nitrogen metabolism-related genes in *StDWF1* overexpression lines. (**A**) Relative expression levels of nitrate reductase (NR) in WT and *StDWF1*-overexpression line (OE2) at 0 h and 3 h. (**B**) Relative expression levels of nitrite reductase (NiR). (**C**) Relative expression levels of aspartate aminotransferase (AT). (**D**) Relative expression levels of nitrate transporter 2.1 (NRT2.1). Data represent means ± SE (*n* = 3). Different letters indicate statistically significant differences according to Student’s *t* test (*p* < 0.05).

Relative expression levels of key nitrogen metabolism-related genes indicated that these genes were significantly up-regulated at 3 h in OE2 compared to WT. For NR, the expression level in OE2 at 3 h was significantly (*p* < 0.05) higher in than WT, showing approximately a 100% increase (Figure 3A). At 0 h, no significant differences were observed between OE2 and WT. For NiR, OE2 exhibited a markedly higher expression level at 3 h compared to WT, with an increase of approximately 188% (Figure 3B). Similar to NR, no differences were observed at 0 h between the two lines. The expression of AT was significantly (*p* < 0.05) upregulated in OE2 at 3 h, with a 190% increase compared to WT (Figure 3C). No significant differences were observed at 0 h between OE2 and WT. For NRT2.1, the expression in OE2 at 3 h was approximately 57% higher than WT (Figure 3D). At 0 h, the expression levels were low and showed no significant differences between the lines.

### 2.4. Improved Nitrogen-Use Efficiency and Stress Tolerance in Transgenic Lines

**Figure 4:**
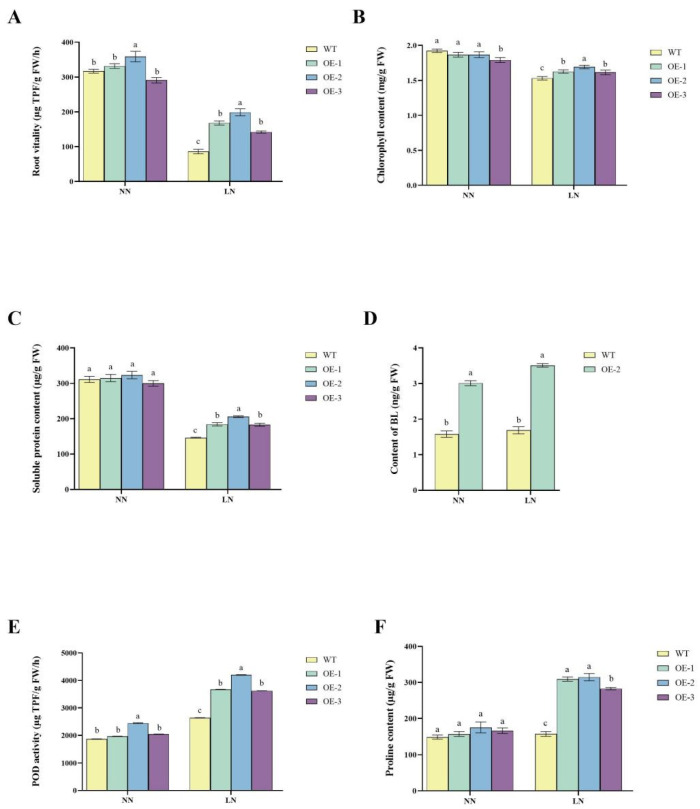
Physiological and biochemical changes in *StDWF1* overexpression lines under varying nitrogen conditions. (**A**) Root vitality of WT and *StDWF1*-overexpression lines (OE1, OE2, OE3) under normal nitrogen (NN) and low nitrogen (LN) conditions. (**B**) Chlorophyll content. (**C**) Soluble protein content. (**D**) Brassinolide (BL) content. (**E**) Peroxidase (POD) activity. (**F**) Protein content. Bars represent means ± SE (*n* = 3). Different letters indicate statistically significant differences according to Student’s *t* test (*p* < 0.05).

OE2 consistently outperformed WT in root vitality, chlorophyll content, soluble protein content, BL content, POD activity, and proline accumulation, demonstrating its superior nitrogen-use efficiency and stress tolerance. Root vitality, as shown in Figure 4, was higher in OE2 under both NN and LN conditions. Under NN, OE2 displayed a 13.2% increase in root vitality compared to WT, while under LN, the improvement was more pronounced, with a 131% higher value than WT (Figure 4A). This indicates that OE2 maintained better root activity and efficiency under nitrogen-deficient conditions. Chlorophyll content remained similar among all lines under NN, but under LN conditions, OE2 retained significantly (*p* < 0.05) higher chlorophyll content compared to WT, showing an increase of 10.5% (Figure 4B). This suggests that OE2 plants exhibited improved chlorophyll retention, which is critical for maintaining photosynthesis under nitrogen stress. The soluble protein content was similar across all lines under NN conditions. However, under LN conditions, OE2 maintained significantly higher (*p* < 0.05) soluble protein levels, showing a 40.6% increase compared to WT (Figure 4C). This suggests that the enhanced ability of OE2 to maintain protein synthesis and stability under nitrogen-deficient conditions contributes to the improvement of low-nitrogen tolerance in potatoes. Additionally, the BL content was significantly higher (*p* < 0.05) in OE2 than in WT under both NN and LN conditions. Under NN conditions, OE2 exhibited a 90% increase in BL content, while under LN conditions, BL levels were approximately 108% higher than in WT (Figure 4D).

Furthermore, POD activity was slightly elevated in the transgenic lines under NN conditions, with OE2 showing a 30% increase compared to WT (Figure 4F). Under LN conditions, POD activity increased significantly (*p* < 0.05) in all lines, with OE2 showing a 59% higher activity compared to WT. Proline content remained similar among all lines under NN conditions. However, under LN conditions, proline content increased significantly (*p* < 0.05) in all lines, with OE2 showing the highest levels, approximately 100% higher than WT (Figure 4E). The elevated proline levels in OE2 suggest improved osmotic regulation and stress adaptation under low nitrogen availability.

### 2.5. Regulation of Hormonal Pathways

**Figure 5:**
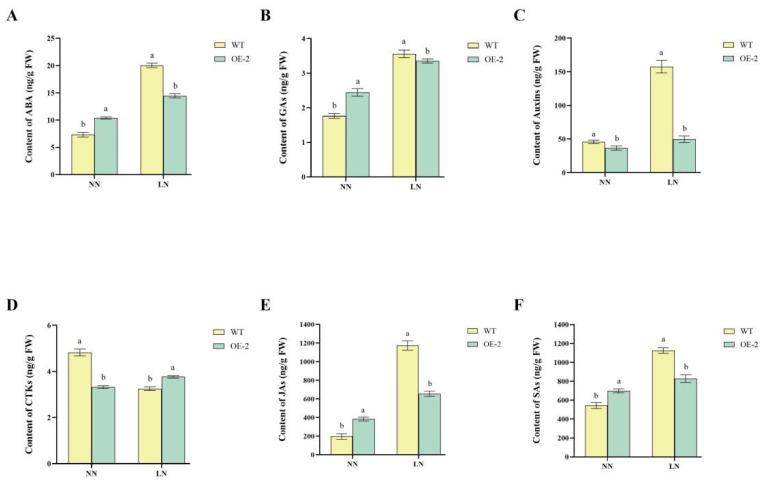
Hormone content in *StDWF1* overexpression lines under varying nitrogen conditions. (**A**) Abscisic acid (ABA) content in WT and *StDWF1*-overexpressing plants (OE2) under normal nitrogen (NN) and low nitrogen (LN) conditions. (**B**) Gibberellins (GAs) content. (**C**) Auxin content. (**D**) Cytokinins (CTKs) content. (**E**) Jasmonic acid (JAs) content. (**F**) Salicylic acid (SAs). Bars represent means ± SE (*n* = 3). Different letters indicate statistically significant differences according to Student’s *t* test (*p* < 0.05).

The levels of most endogenous hormones responded dynamically to LN stress. Under LN conditions, the levels of ABA, GA, JA, and SA increased in both OE2 and WT, though the accumulation in WT was significantly higher than in OE2. Specifically, under NN conditions, OE2 exhibited significantly higher ABA levels than WT. Under LN stress, ABA levels increased in both lines, but WT accumulated significantly higher ABA content than OE2 (Figure 5A). OE2 consistently exhibited higher GA levels than WT under both NN and LN conditions, though the difference was more pronounced under NN conditions. Under LN stress, GA content increased in both OE2 and WT, but WT exhibited a significantly higher GA accumulation than OE2, indicating that WT may activate GA-mediated growth responses more strongly under nitrogen limitation (Figure 5B). IAA levels in WT were significantly higher than in OE2 under both NN and LN conditions, with an even greater accumulation under LN stress (Figure 5C). Under NN conditions, OE2 exhibited significantly lower CTK levels than WT. However, under LN stress, CTK levels in WT declined sharply, while in OE2, they remained relatively stable, leading to higher CTK levels in OE2 than in WT under LN conditions (Figure 5D). JA levels were significantly higher in OE2 compared to WT under both NN and LN conditions. However, under LN stress, WT exhibited a significantly higher increase in JA content compared to OE2, suggesting that JA accumulation is a major component of the WT stress response (Figure 5E). Under NN conditions, OE2 exhibited significantly lower SA levels than WT. After LN stress, SA levels increased in both lines, but WT had significantly higher SA accumulation than OE2, reinforcing the idea that WT relies on stronger SA-mediated defense mechanisms in response to nitrogen limitation (Figure 5F).

### 2.6. Dynamic Regulation of StDWF1, StGRP1 Expression

**Figure 6:**
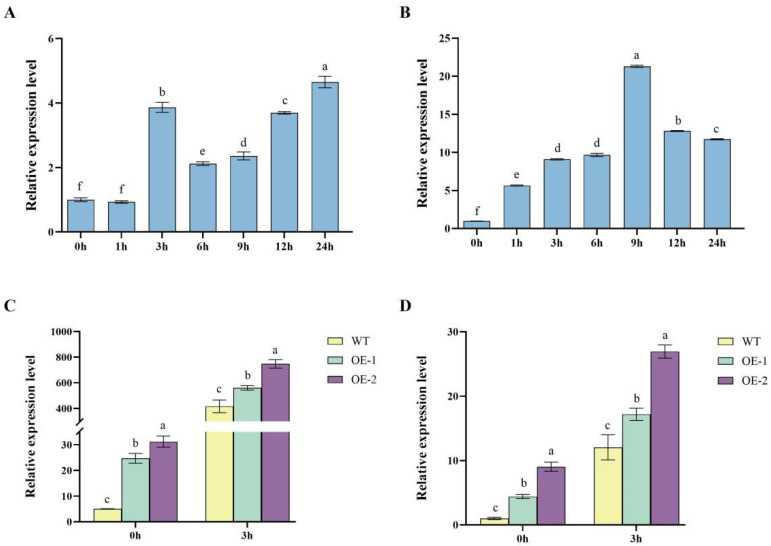
Temporal and genotypic expression profiles of *StDWF1* and stress-related genes in *StDWF1* overexpression lines. (**A**) Time-course analysis of *StDWF1* relative expression levels in WT plants under stress conditions. (**B**) Time-course analysis of *StGRP1* relative expression levels. (**C**) Comparative expression levels of *StDWF1* in WT and overexpression lines (OE1, OE2) at 0 h and 3 h after stress induction. (**D**) Relative expression of *StGRP1*. Bars represent means ± SE (*n* = 3). Different letters indicate statistically significant differences according to Student’s *t* test (*p* < 0.05).

The temporal expression analysis of *StDWF1* and *StGRP1*, together with their relative expression in WT, OE1 and OE2, provides compelling evidence for the dynamic regulation of these genes under specific conditions. In the temporal analysis, *StDWF1* expression gradually increased over time, reaching its peak at 24 h with a 3.7-fold increase compared to the baseline at 0 h (Figure 6A). The expression of *StGRP1* followed a similar trend, peaking at 9 h with a 20.3-fold increase compared to 0 h, before declining by 24 h (Figure 6B).

When comparing relative expression levels between WT and the transgenic lines, significant differences were observed. For *StDWF1*, both OE1 and OE2 showed dramatically higher expression levels compared to WT, particularly at 3 h. OE2 exhibited the highest expression, which was approximately 79% higher than WT, while OE1 displayed a 34.4% increase (Figure 6C). Similarly, *StGRP1* expression was significantly (*p* < 0.05) higher in OE2 at 3 h, showing a 124% increase compared to WT, while OE1 showed a 43% increase (Figure 6D).

### 2.7. Validation of StDWF1 and StGRP Interactions Using Yeast Two-Hybrid and BiLC Assays

**Figure 7:**
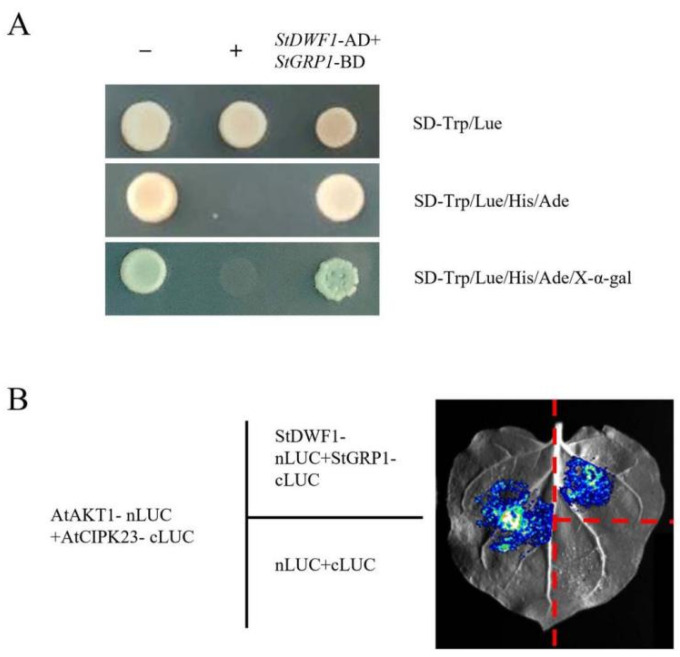
Protein–protein interaction analysis of *StDWF1* and *StGRP1*. (**A**) Interaction between *StDWF1* and *StGRP1* was assessed using Y2H analysis. Growth of yeast transformants on selective media is shown: SD-Trp/-Leu (non-selective control media). SD-Trp/-Leu/-His/-Ade (selective media). SD-Trp/-Leu/-His/-Ade/X-α-gal (highly selective media with X-α-gal for interaction visualization). Positive interactions are indicated by growth and blue color development. Yeast expressing *StDWF1* with *StGRP1* shows robust growth and blue color, confirming protein–protein interactions. (**B**) A schematic representation of BiFC analysis setup. Protein pairs tested: AtAKT1+AtCIPK23 (positive control), nLUC+cLUC (negative control), *StDWF1+StGRP1*. Leaf infiltration of tobacco (*Nicotiana benthamiana*) showed luciferase reconstitution and interaction between *StDWF1* and *StGRP1*. Luminescence imaging showed a strong interaction signal between *StDWF1* and *StGRP1*.

The yeast two-hybrid assay provided robust validation of interactions between StDWF1 and StGRP1, demonstrating their potential functional relevance (Figure 7A). Yeast strains co-transformed with *StDWF1-AD+StGRP1-BD* showed strong growth on highly selective media (SD-Trp/-Leu/-His/-Ade/X-α-gal), confirming that StGRP1 specifically interacts with StDWF1. In contrast, the negative controls (*pGADT7+pGBKT7* and *StCBL4-AD+StCIPK23-BD*, first and fourth columns, respectively) showed negligible or no growth on the same selective media, demonstrating that the observed growth in the test groups is specific to the interaction of StDWF1 with StGRP1.

The BiLC assay further corroborates these findings and highlights the functional interaction between *StDWF1* and *StGRP1* in planta (Figure 7B). The BiLC assay utilized complementary luciferase fragments (nLUC and cLUC) fused to candidate interacting proteins, where luminescence signals indicated physical interactions between the protein pairs. As a positive control, the interaction between *AtAKT1* and *AtCIPK23* resulted in strong luminescence, validating the experimental setup. Similarly, the interaction between StDWF1 and StGRP1 produced a detectable luminescence signal. In contrast, the negative control (nLUC+cLUC; lower right quadrant) showed no luminescence, confirming the specificity of the observed interactions and ruling out nonspecific complementation of luciferase fragments.

## 3. Discussion

Improving the low-nitrogen resistance of plants is a fundamental goal of crop biotechnology, especially in the context of sustainable agriculture and environmental protection. Nitrogen influences key physiological and biochemical processes such as chlorophyll synthesis, protein production, and enzyme activation [28]. However, low nitrogen affects normal plant growth and development, and overuse of fertilizers can lead to environmental degradation and economic challenges [28,29]. A deeper understanding of the mechanisms of low nitrogen stress in plants can help develop crop varieties that thrive under nitrogen-limited conditions while reducing ecological impacts. This study focused on the transgenic OE2 potato line, which expressed 31-fold more *StDWF1* than the WT line. Identification of *StDWF1* at 24 h after exogenous BR treatment showed that *StDWF1* responded to exogenous BR regulation, peaking at 3 h after induction. Under LN conditions, *StDWF1* overexpression lines grew better than WT, as evidenced by higher plant height and longer root system of the overexpression lines. The metabolism-related physiological data indicated that the overexpression lines increased plant nitrogen accumulation by enhancing the nitrogen uptake and assimilation capacity of the plants. The observed increase in nitrogen assimilation efficiency in OE2 may be attributed to BR signaling, which has been shown to regulate nitrogen uptake by modulating the expression of transporter genes. BR treatment has been shown to induce the expression of NH_4_^+^ transporters *AMT1.1* and *AMT1.2* in rice through the transcription factor ABI3/VP1-Like 1 (RAVL1), thereby enhancing NH_4_^+^ uptake [30]. Overexpression of StDWF1 may have stimulated BL biosynthesis and thus induced the expression of NH4+ transporter genes, resulting in high OE2 ammonium uptake capacity. Additionally, previous research in cucumber under LN conditions has shown that exogenous BL treatment enhances the expression of key nitrogen metabolism enzyme genes [31]. Similarly, in maize, BRs regulate nitrogen responses by modulating the transcription of *NRT* genes [32], further supporting the hypothesis that BR biosynthesis is a critical factor in nitrogen absorption. Consistent with these findings, our study revealed that, before LN treatment, the relative expression levels of *NR* and *NiR* were comparable between WT and OE2. However, after LN treatment, both genes were significantly upregulated in OE2, along with *AT* and *NRT2.1* [32,33]. This suggests that *StDWF1* may influence nitrogen uptake and assimilation by promoting BR biosynthesis, further reinforcing the connection between BR signaling and nitrogen metabolism. Together, these findings suggest that overexpression of the StDWF1 strain utilizes a BR-mediated mechanism to enhance N uptake and assimilation in potatoes, thereby improving resistance and metabolic stability under nitrogen-limited conditions. Additionally, OE2 showed a 43% increase in root vitality and 23% higher chlorophyll retention under LN conditions, indicating greater metabolic stability and improved stress resilience [34].

OE2 plants demonstrated the ability to retain chlorophyll under nitrogen-deficient conditions, allowing them to sustain photosynthesis and growth [35]. Maintaining chlorophyll content is essential for energy production and overall plant health [36]. Despite the significant upregulation of *StDWF1*, chlorophyll levels remained comparable across all lines under NN conditions, suggesting that chlorophyll synthesis may not be a limiting factor for photosynthetic efficiency under optimal nitrogen supply [37]. Under LN conditions, OE2 exhibited a 25% increase in soluble protein levels, a 35% rise in POD activity, and a 45% increase in proline accumulation. These enhancements suggest improved cellular stability, better oxidative stress mitigation, and enhanced osmotic balance [38,39]. The higher soluble protein content in OE2 likely contributes to increased enzymatic activity, which is crucial for metabolic adaptation in nitrogen-deficient environments [40]. The elevated POD activity further indicates a stronger antioxidant defense system, reducing oxidative stress and preserving cellular integrity [41]. Additionally, higher proline accumulation in OE2 acts as an osmoprotectant, stabilizing proteins and membranes while minimizing oxidative damage, thereby enhancing plant survival under stress [39]. Phenotypic assessments revealed that OE2 outperformed WT in both shoot and root growth under NN and LN conditions. Specifically, OE2 showed a 19% increase in root vitality under NN and an even greater 43% increase under LN conditions. The more pronounced improvement under nitrogen-limited conditions suggests that *StDWF1* overexpression provides adaptive advantages during nitrogen stress [42]. Enhanced root development under stress is particularly significant, as a strong root system promotes efficient nutrient and water uptake, reinforcing plant resilience in suboptimal environments [34].

The endogenous oleuropein lactone content detected in the overexpressed StDWF1 strain was higher than that of WT, and the synthesis of BR was induced by low nitrogen. Overexpressing strains significantly up-regulated the *StDWF1* gene under low-nitrogen conditions, and overexpression of the gene promoted the synthesis of oleuropein lactone (BL) and increased the endogenous content of BL. When phytohormones regulate plant growth and development, there are complex interactions, collaborative or antagonistic, between hormones involved in the plant response to nitrogen availability. Previous studies have found that nitrogen effectiveness influences the levels of hormones associated with growth and stress responses [43]. Stress regulatory mechanisms are rapidly generated in plants under low-nitrogen adversity that regulate the levels of nitrogen-related hormones and directly affect the plant’s ability to adapt to low-nitrogen stress [44]. These findings were similar to the results of hormone content measurements in this experiment. The contents of stress hormones ABA, JA and SA in OE2 and WT increased significantly in response to low-nitrogen stress, and the contents of stress hormones in WT were higher than those in OE2 due to the higher BL content of OE2 which improved plant resistance, and the WT was affected by low-nitrogen stress more than that in OE2. And low nitrogen induced a rise in GA content in WT, which also promoted the increase in IAA content. The CTK content was higher in OE2 than in WT after low nitrogen stress, which may be due to the fact that OE2 grows better under low nitrogen conditions and has a more developed root system, which results in higher CTK synthesis.

*OsGSR1* (Oryza sativa GA-Stimulated Transcript 1) is an important gene in rice belonging to the GAST (GA-Stimulated Transcript) gene family, which regulates growth, development and yield traits by mediating the crosstalk between gibberellin and oleuropein lactone signaling pathways [45]. We selected a gene in the potato with the highest homology to *OsGSR1* in the GAST family, named *GRP1*. We investigated their effects on plant stress tolerance by studying their interactions with *DWF1* protein. The strong interaction between *StDWF1* and *StGRP1* suggests that *StGRP1* may influence the function or stability of *StDWF1* to regulate downstream processes related to the response to low nitrogen stress. The results of LN treatment for 24 h showed that *StGRP1* was as involved in the low nitrogen response as *StDWF1*. In order to investigate whether *StDWF1* and *StGRP1* respond to low-nitrogen stress separately or participate in the LN response together through protein interactions, we examined the expression levels of their genes under low-nitrogen conditions, and the results showed that *StDWF1* and *StGRP1* shared a similar expression pattern, so it was hypothesized that there was a tendency for *StGRP1* to be co-expressed with *StDWF1*. Wang et al. showed that *OsGSR1-RNAi* lines exhibited typical characteristics of BR-deficient plants with reduced endogenous BR levels, and that *OsGSR1* interacted with *DIM/DWF1* to promote the synthesis of BRs and regulate plant growth and development [45]. Similarities were also found in Nahirñak et al. where Snakin-1-silenced lines showed phenotypic alterations and enhanced GA levels typical of BR-deficient mutants, and a large number of genes related to sterol biosynthesis were detected to be down-regulated in the silenced lines [46]. Higher *StGRP1* expression was detected in OE lines than in WT and was significantly and positively correlated with the expression of *StDWF1*, which was also subjected to low nitrogen-induced expression, and the content of endogenous BL in the plants coincided with the gene expression levels. The endogenous BL content of the plants matched the gene expression level, and *StGRP1* interacted with *StDWF1*. These results suggest that *StGRP1* may regulate phytosterol biosynthesis and positively promote the biosynthesis of BRs by interacting with *StDWF1*, and that this ability will be induced by low-nitrogen stress, altering changes in endogenous hormone content and regulating the plant’s adaptation to the low-nitrogen environment.

This study confirmed the correlation between *StDWF1* overexpression and low-nitrogen tolerance in the potato, but some limitations still exist in the experiment. There is a clear correlation between up-regulation of gene transcript levels and phenotypic changes; however, existing experiments cannot fully explain whether exogenous genes are integrated into the potato or not, while changes in the expression levels of proteins, as the direct executors of cellular functions, are closely related to the phenotypes of organisms. In addition, it is important to note that the carrier system itself may cause the test to be biased. Therefore, in future studies, the identification of transgenic strains and protein level studies should be strengthened to clarify the function of *StDWF1*. At the same time, empty vector lines should be constructed to eliminate the interference of the vector in the experiment.

## 4. Materials and Methods

### 4.1. Generation of StDWF1 Overexpression Transgenic Potato Lines

The wild-type material used in this experiment was the potato variety “Chuanyu 10”. To generate StDWF1 overexpression (OE) transgenic lines, an Agrobacterium-mediated transformation method was employed [47], using “Chuanyu 10” as the transformation material. The transformation vector, which contained the StDWF1 gene, a key player in brassinolide biosynthesis, was introduced into potatoes under the control of the CMV35S promoter. As a result, transgenic potato plants with enhanced StDWF1 expression were successfully developed.

### 4.2. Hydroponic Cultivation of Seedlings and Phenotypic Observation

WT and OE potato seedlings grown on normal MS medium for 25 days were selected. The apical tips of the seedlings (about 1.5–2 cm) were excised under a laminar flow hood and transferred to normal MS (Murashige and Skoog) medium in test tubes for 7 days. Each culture tube contained 10 plant segments. The seedlings were cultivated in a growth chamber at 23 ± 2 °C with a 16 h light/8 h dark cycle and light intensity of 3000 lx. After 7 days, roots began to develop. The seedlings were then removed from the medium, rinsed to remove any residual medium, and transferred to a normal MS hydroponic solution (containing 30 mM nitrogen) and low-nitrogen MS hydroponic solution (containing 7.5 mM nitrogen) for hydroponic treatment. The culture conditions remained unchanged, with no added sucrose or agar, and the pH was adjusted to 5.8. Phenotypic observations were recorded after 20 days of hydroponic cultivation, including photographs and phenotype measurements. Three biological replicates were used per treatment.

### 4.3. Nitrogen Depletion Experiment

WT and OE seedlings were grown on normal solid MS medium for 7 days and then hydroponically grown in 5-fold diluted liquid MS medium for 12 days. The seedlings, showing consistent growth, were then treated with 0.2 mM CaSO_4_ solution for 48 h as the starvation treatment. Afterward, 3–5 seedlings that exhibited consistent growth after nitrogen starvation treatment were placed into 1 mM KNO_3_ solution (pH 5.8–6.0) in conical flasks, and cultured in a shaker at 100 rpm. Samples were taken at 0, 2, 4, 6, and 8 h. For sampling, 1 mL of the depletion solution from the center of the conical flask was collected at each time point and stored in 1.5 mL centrifuge tubes at 4 °C. After sampling, an equal volume of ultrapure water was added to maintain the volume of the depletion solution. The nitrate nitrogen concentration in the samples was determined using the nitrate nitrogen assay kit from GRI Science. Three biological replicates were used per treatment.

### 4.4. Enzyme Activity Assay

Potato seedlings from the nitrogen depletion experiment continued to receive treatment in the nitrogen depletion solution for 20 days, after which whole plants were taken and stored in liquid nitrogen, with three plants per treatment. The samples were taken as 0.1 g by grinding the whole plant using a mortar and pestle. Nitrate and ammonium nitrogen levels were measured using GRI Science’s Nitrate and Ammonium Nitrogen Assay Kits. The activity of key nitrogen metabolism enzymes, nitrate reductase (NR) and glutamine synthetase (GS), was measured using enzyme activity assay kits from Solarbio Biotechnology (Shanghai, China). Each treatment had three biological replicates and technical replicates.

### 4.5. Determination of Plant Hormones and Physiological Indicators

The WT and OE histocultures were removed after 7 d of growth in normal MS medium, washed to remove residual medium on the roots, and then subjected to 20 d of hydroponic treatment with prepared normal MS medium (containing 30 mM N) and low nitrogen MS medium (containing 7.5 mM N) under the same culture conditions. Fresh samples of whole plants were collected and stored in liquid nitrogen, and then sent to the company (Nanjing Ruiyuan Biotechnology Co., Nanjing, China) to analyze the content of seven hormone types, namely oleuropein lactones (BL), gibberellins (GAs), abscisic acid (ABA), cytokines (CTKs), growth hormones (auxins), jasmonic acids (JAs) and salicylic acids (SAs). Each treatment was replicated three times.

Leaves of potato hydroponic seedlings were collected and extracted with acetone-ethanol mixture to determine chlorophyll content [48]; soluble protein content was determined by the G-250 method [49]; peroxidase (POD) activity was determined by the guaiacol method [50]; proline content was determined by the acid ninhydrin method [51]; and roots of potato hydroponic seedlings were collected in detail, with root vitality determined by the triamcinolone tetrazolium chloride (TTC) method [52]. Three treatments per replicate were used.

### 4.6. LUC Assay

StDWF1-JW772 and StGRP1-JW772 recombinant vectors were sent to Wuhan Jinkairui Bioengineering Co., Ltd., Wuhan, China. The recombinant StDWF1-JW772 and StGRP1-JW772 vectors were transformed into Agrobacterium GV3101. The transformed Agrobacterium GV3101 was cultured overnight at 28 °C with shaking at 200 rpm in root-nodule Agrobacterium liquid medium (YEB) to an optical density (A600) of 0.5–1.0. Agrobacterium suspensions were mixed in equal volumes using an infestation buffer containing 10 mM MES (2-morpholine ethanesulfonic acid), 0.2 mM Acetosyringone, and 10 mM MgCl₂ fractions, and the OD₆₀₀ value was adjusted to 1.0. After that, the mixed bacterial solution was incubated at 28 °C incubator for 2–3 h to allow the bacteria to adapt to the buffer environment. The Agrobacterium suspension was used to infect tobacco leaves (approximately 4 weeks old), which were then incubated in the dark at 25 °C for 24 h. LUC activity was measured using a chemiluminescence imaging system (Tanon 5200 Multi, Shanghai, China). In the experiment, *AtAKT1+AtCIPK23* was used as the positive control, and nLUC+cLUC was used as the negative control.

### 4.7. Yeast Two-Hybrid Assay

The coding region of the StDWF1 gene was inserted into the pGADT7 vector using NdeI and BamHI enzymes, and the coding region of the StGRP1 gene was inserted into the pGBKT7 vector. The recombinant vectors were transformed into yeast AH109 cells using the PEG/LiAc method, and the successfully transformed colonies were screened after 2–4 d of growth at 28 °C in SD-Leu-Trp medium. Yeast growth on SD-Trp-Leu-His-Ade medium was then observed. The negative control was pGADT7+pGBKT7 and the positive control was AtCBL1+AtCIPK23. The experiment was repeated three times.

### 4.8. RNA Extraction and qPCR Identification

The WT and OE seedlings were removed after 20 d of growth in normal MS medium and then treated hydroponically with prepared normal MS medium (containing 30 mM N) and low-nitrogen MS medium (containing 7.5 mM N) under the same culture conditions. Whole plant samples were collected at 0 h and 3 h to measure the relative gene expression of NR, NiR, AT, NRT2.1, StDWF1, and StGRP1. Whole plant samples were collected at 0 h, 1 h, 3 h, 6 h, 9 h, 12 h, and 24 h to measure the relative gene expression of StDWF1 and StGRP1.

WT seedlings were grown in normal MS medium for 20 d, then transferred to MS medium containing 500 nM BL for 24 h under the same culture conditions, and whole plant samples were collected at 0 h, 1 h, 3 h, 6 h, 9 h, 12 h, and 24 h to measure the relative gene expression of StDWF1.

All samples were immediately frozen in liquid nitrogen and stored at −80 °C. Total RNA was extracted using the TRIzol method, and the extracted RNA was reverse transcribed into cDNA using the Reverse Transcription Kit (RT mix with DNase) from Suzhou Yuheng Biotechnology Co., Ltd., Suzhou, China. Real-time quantitative PCR was performed using a fluorescent quantitative enzyme (SYBRPRIME qPCR Kit) from Bioground Biologicals (Chongqing, China), and StEF-1α was used as the reference gene. The relative gene expression of each gene was calculated using the 2^−ΔΔCT^ method, and all reactions were subjected to three biological and technical replicates (Table S1).

### 4.9. Statistical Analysis and Methods 

Significance of qPCR data from three independent biological replicates was analyzed using the SPSS 27 statistical software Student’s *t* test, with the significance level set at *p* < 0.05. Bar charts were created using the GraphPad Prism 10.3 software.

## 5. Conclusions

In this study, we found that the overexpressed *StDWF1* lines grew better than WT under LN conditions, with higher chlorophyll, proline, and soluble protein contents, and stronger root vigor and POD enzyme activity. The nitrogen depletion test showed that OE strain had stronger nitrogen uptake and assimilation capacity to increase plant nitrogen accumulation, and the nitrogen transporter genes and nitrogen metabolism key enzyme genes were significantly up-regulated in OE strain after LN treatment, which indicated that *StDWF1* was able to improve the nitrogen utilization efficiency of potato under low-nitrogen conditions. The results of the hormone content assay showed that the endogenous oleuropein lactone (BL) content of *StDWF1* overexpression lines was significantly increased under LN conditions, while LN also induced a rise in abscisic acid (ABA), gibberellin (GAs), growth hormones (Auxins), jasmonic acid (JAs), and salicylic acid (SAs) content. The yeast two-hybrid assay and luciferase complementation assay demonstrated the existence of protein interactions between *StGRP1* and *StDWF1*, and the qRT-PCR results showed that *StGRP1* and *StDWF1* shared similar expression patterns. In summary, overexpression of *StDWF1* improved the tolerance of transgenic potato lines under low nitrogen stress.

## Data Availability

The data presented in this study are available within the article.

## Supplementary Materials

The following supporting information can be downloaded at https://www.mdpi.com/article/10.3390/ijms26094374/s1.
